# How nesting time affects the health and survival of migratory birds

**DOI:** 10.1093/conphys/coaf065

**Published:** 2025-09-09

**Authors:** Mobassher Hossain

**Affiliations:** Department of Fisheries, University of Rajshahi, Rajshahi 6205, Bangladesh

Franklin’s Gulls (*Leucophaeus pipixcan*), charismatic long-distance migrants of the Northern Great Plains of Canada and the USA, may appear to be synchronized symbols of spring, but beneath the surface of their bustling breeding colonies lie subtle, critical differences that may shape the survival of the species.

Chicks from early-season nests of Franklin’s Gulls grow larger and have better survival outcomes. So why do some of the birds nest late? Are they unhealthy? Disruptions in food availability, heightened competition and physiological stressors may be placing late-nesting birds at a disadvantage. A recent study by Shawn Weissenfluh and colleagues (Weissenfluh *et al*., 2025) sheds light on the relationship between the timing of nesting and health, stress responses and immune function in the nesting gulls.

To investigate, the team captured 61 adults, nesting gulls (Fig. 1) in North Dakota during the breeding season, which spanned from May to mid-June and assessed their physiological state using three health indicators: residual body mass (to evaluate energy reserves), corticosterone levels (a hormone that quickly increases during acute stress) and white cell counts and bacterial-killing ability of blood plasma (to assess immune system strength).

**Figure 1:**
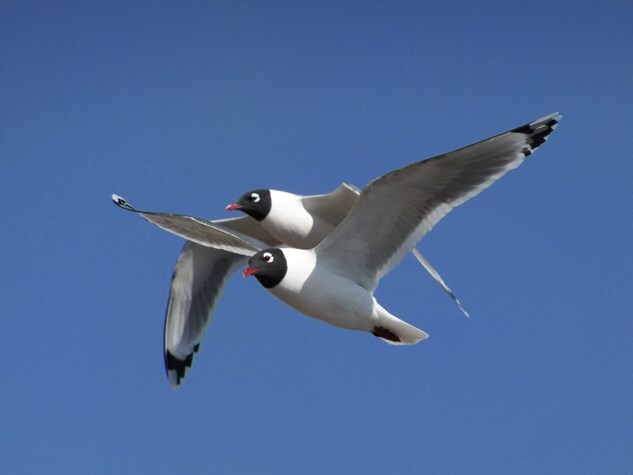
Pair of Franklin’s gulls in flight. Image credit: Shawn Weissenfluh

The results were striking. Gulls nesting later in the season were in noticeably poorer condition. They had less flight muscle mass—evident by lower body mass relative to size and breastbones, which stuck out above the breast muscles. They had an exaggerated reaction to stress, with elevated corticosterone levels after capture and handling. Most critically, the later nesters had weaker immune function, shown by a reduced ability of their plasma to kill bacteria in laboratory tests.

These physiological differences were not related to sex or time of day when data were collected, but were related unequivocally to when each bird nested during the breeding season. Later-nesting individuals were compromised, though whether it was that compromise that led them to breeding late is an open question. Whatever its origin, late nesting disadvantaged the chicks, perhaps due to declining food availability or less time to prepare for their return migration.

So why does timing matter, as long as enough gulls nest early? In migratory species like Franklin’s Gulls, nesting seasons are brief, and any delay can force birds to balance staying healthy with raising their young. As global temperatures rise and nesting times change, more birds may end up nesting late, potentially, weakening population health over time.

Ultimately, Weissenfluh and colleagues offer a valuable framework for studying how subtle shifts in seasonal timing ripple through wild bird physiology. Their work underscores the need for conservation strategies that buffer vulnerable populations from these shifts—such as protecting key stopover habitats, ensuring consistent food resources, and monitoring population health throughout the breeding season.

As climate change continues to reshape the rhythms of nature, understanding and mitigating its impacts on migratory birds is not just important science—it is conservation imperative.

## Notes

**Editor:** Ashleigh Donaldson
